# Efficacy and safety of cadonilimab for malignant solid tumor treatment: a systematic review and meta-analysis

**DOI:** 10.3389/fimmu.2026.1851837

**Published:** 2026-05-29

**Authors:** Xiaodong Mi, Tong Lin, Xiaogang Zhu, Li Tian, Juntao Liu, Hong Qin, Shimin Hou, Fei Tuo

**Affiliations:** 1 Department of Obstetrics and Gynecology, People’s Hospital of Xiangxi Tujia and Miao Autonomous Prefecture, First Affiliated Hospital of Jishou University, Jishou, Hunan, China; 2 Department of Obstetrics and Gynecology, The Third Xiangya Hospital of Central South University, Changsha, Hunan, China

**Keywords:** bispecific antibody, cadonilimab, CTLA-4, ICIS, PD-1, solid tumor

## Abstract

**Objective:**

To evaluate the efficacy and safety of cadonilimab in patients with solid tumors.

**Methods:**

We systematically searched seven databases—PubMed, Embase, Web of Science, Cochrane Library, Ovid MEDLINE, Scopus, and ProQuest—for clinical studies published up to July 19, 2025. Inclusion criteria encompassed randomized controlled trials (RCTs) and single-arm trials. Data collected included objective response rate (ORR), disease control rate (DCR), overall survival (OS), progression-free survival (PFS), and adverse events (AEs) incidence. A meta-analysis of relevant data was performed using a random-effects model.

**Results:**

This study encompassed a total of 13 investigations, comprising 11 single-arm clinical trials, and 2 randomized controlled trials. A total of 1,359 patients with solid tumors, including gastric cancer or adenocarcinoma of the gastroesophageal junction and cervical non-small cell lung cancer, were enrolled in the study. The pooled efficacy analysis demonstrated an ORR of 0.45 (95% CI: 0.31–0.59), a DCR of 0.84 (95% CI: 0.71–0.94), a median PFS of 7.47 months (95% CI: 4.98–9.97), and a median OS of 12.89 months (95% CI: 10.09–15.68). Subgroup analysis indicated the highest ORR in cervical cancer (0.63, 95% CI: 0.33–0.94), while the highest DCR was observed in gastric or gastroesophageal junction adenocarcinoma (0.91, 95% CI: 0.82–1.00). The administration of cadonilimab at a dosage of 10 mg/kg every three weeks resulted in the optimal ORR and DCR of 0.71 (95% CI: 0.63–0.80) and 0.95 (95% CI: 0.90–0.99), respectively. A comparison of cadonilimab plus chemotherapy with monotherapy or combination therapy with targeted agents revealed that the former demonstrated superior efficacy, with an ORR and DCR of 0.62 (95% CI: 0.56–0.69) and 0.94 (95% CI: 0.88–1.00), respectively. With respect to safety, the incidence rate of adverse events of any grade was 0.99 (95% CI: 0.98–1.00), the incidence of ≥Grade 3 treatment-related adverse events (TRAEs) was 0.49 (95% CI: 0.34–0.63), and the incidence of ≥Grade 3 immune-related adverse events (irAEs) was 0.11 (95% CI: 0.08–0.14). The most prevalent TRAEs included neutrophil count decreased, anemia, and platelet count decreased.

**Conclusions:**

Cadonilimab demonstrates a positive response in the treatment of various solid tumors, exhibiting good tolerability.

**Systematic review registration:**

https://www.crd.york.ac.uk/prospero/, identifier CRD420251103576.

## 1 Introduction

Immunotherapy has emerged as a significant treatment option alongside surgery, radiation therapy, chemotherapy, and targeted therapy, offering new strategies for the treatment of malignant tumors (1). The Food and Drug Administration (FDA) has now approved these types of immune checkpoint inhibitors (ICIs) to treat tumors: anti-PD-1 inhibitors, anti-PD-L1 inhibitors, anti-CTLA-4 inhibitors (2–4). These anticancer drugs are predominantly monoclonal antibody (mAb), primarily exerting their effects by blocking specific targets such as programmed death receptor-1 (PD-1) and cytotoxic T-lymphocyte-associated antigen-4 (CTLA-4) (5). However, throughout the course of anticancer treatment, such agents (e.g., PD-L1 inhibitors) may provoke tumor resistance, as cancer cells and immunosuppressive cells inside the tumor microenvironment exhibit additional inhibitory checkpoints beyond PD-L1 (5, 6). Furthermore, the efficacy of mAb is contingent on the body’s pre-existing immune response, rendering them less effective in tumors with low immunogenicity or a weak initial immune response (7). To address the limited effectiveness of mAb, clinical practice frequently uses combination therapy involving several mAb. This strategy enhances treatment response rates but concurrently raises the occurrence of adverse effects (AEs) (8). mAb treatment can disturb the equilibrium between inhibitory and co-stimulatory signals in normal tissues, resulting in heightened immune system activation and an increased incidence and severity of immune-related adverse events (irAEs) (9). These factors collectively propel the advancement of multi-target pharmaceuticals, including bispecific antibodies (bsAbs).

Cadonilimab (AK104), the inaugural PD-1/CTLA-4 bispecific antibody to receive approval (10), transcends conventional constraints via its novel tetravalent architecture and Fc-null configuration. By concurrently engaging the PD-1 and CTLA-4 pathways, it effectively disinhibits PD-1-mediated T-cell “immune brakes” and eradicates CTLA-4-positive immunosuppressive Treg cells. This dual approach synergistically improves T-cell activation and infiltration in the tumor microenvironment (11). Fundamental studies verify that it demonstrates increased binding affinity in tumor microenvironments characterized by elevated PD-1/CTLA-4 density. Its deficiency in Fc receptor binding capacity markedly diminishes antibody-dependent cellular cytotoxicity (ADCC) and cytokine release (e.g., IL-6/IL-8). This medicine theoretically has specific therapeutic efficacy and safety (11–14). The early Phase I trial, COMPASSION-01, further validated that it achieved an ORR of 13.4% in advanced solid tumors, with a median duration of response (DoR) of 12.9 months. Furthermore, the incidence of irAEs was found to be significantly lower in comparison to that observed with PD-1/CTLA-4 monoclonal antibody combination regimens (15), thus providing both theoretical and clinical feasibility for bsAbs immunotherapy.

The therapeutic efficacy of cadonilimab has been confirmed across several tumor types. Cadonilimab attained a significant advancement in cervical cancer by combination therapy: The Phase III COMPASSION-16 trial (16) indicated that the combination of platinum-based chemotherapy ± bevacizumab as first-line treatment for recurrent/metastatic cervical cancer (r/mCC) resulted in a median progression-free survival (mPFS) of 12.7 months (compared to 8.1 months in the placebo group), with an ORR surpassing 90%. Survival advantages were also noted in PD-L1-negative individuals. Consequent to these findings, China’s National Medical Products Administration (NMPA) incorporated it into its 2022 treatment guidelines for recurrent or metastatic cervical cancer subsequent to progression following platinum-based chemotherapy (10). In the field of liver cancer, the Phase II COMPASSION-08 trial demonstrated that the combination of lenvatinib as first-line therapy for unresectable hepatocellular carcinoma (uHCC) achieved an ORR of 35.5% and a median overall survival (mOS) of 27.1 months, offering a new option for targeted therapy combined with immunotherapy (17). The Phase III COMPASSION-15 trial demonstrated that the combination of cadonilimab and chemotherapy resulted in an ORR of 65.2% (compared to 48.9% in the chemotherapy-only group) for the first-line treatment of HER2-negative advanced gastric or gastroesophageal junction adenocarcinoma. The trial also demonstrated significantly prolonged mOS to 14.1 months and maintained efficacy even in the low PD-L1 CPS < 5 expression subgroup (18). With regard to the combination with anti-angiogenic agents, the Ib/II phase trial with anlotinib for advanced non-small cell lung cancer (NSCLC) achieved an ORR of 60%, with a treatment-related adverse event (TRAE) incidence of grade 3 or higher recorded in only 25% of cases (19). Nonetheless, bsAbs medications are associated with the potential for irAEs, including infusion responses (20), allergic reactions (21), and, in rare cases, toxic epidermal necrolysis (22). Furthermore, existing safety data for combination therapy are predominantly dispersed throughout small-sample trials focused on individual tumor types, missing comprehensive assessment across treatment regimens and extensive research, hence creating ambiguity for clinical application.

Notwithstanding the accumulation of considerable preliminary data for cadonilimab monotherapy and combination therapy, important information gaps remain in the existing study. Initially, comparative efficacy data are insufficient—data concerning cervical cancer, gastric cancer, and other tumor types predominantly stem from Phase III trials (16, 18), whereas hepatocellular carcinoma (19), nasopharyngeal carcinoma (23), and additional tumor types are largely characterized by single-arm studies. No comparative data exists about the efficacy of cadonilimab across various tumors or among specific patient subgroups. Secondly, safety evaluations are insufficient—current research primarily emphasizes isolated AEs (e.g., allergic reactions, infusion reactions) and neglects to measure the overall prevalence of common iirAEs (e.g., thyroid dysfunction, pneumonia) and severe AEs (≥Grade 3) (20, 21). Although research indicates that combined chemotherapy and targeted therapy may enhance overall response rates, the trade-off between efficacy improvements and heightened toxicity compared to monotherapy is still ambiguous (17, 18). This study aims to perform a systematic review and meta-analysis to: first, summarize the overall efficacy of cadonilimab monotherapy and combination therapy across various solid tumors and compare their efficacy among different subgroups; second, quantify the incidence rates of common irAEs and high-grade AEs, establish a safety profile, and offer evidence-based guidance for clinical treatment selection. This study’s findings aim to address existing evidence gaps, inform personalized treatment decisions, and offer guidance for future clinical trial designs.

## 2 Materials and methods

The present study adhered to the Preferred Reporting Items for Systematic Reviews and Meta-Analyses (PRISMA) criteria (24). Furthermore, the meta-analysis protocol was registered with PROSPERO (NO: CRD420251103576).

### 2.1 Literature searching strategies

We conducted a systematic search across seven databases: PubMed, Embase, Web of Science, Cochrane Library, Ovid MEDLINE, Scopus, and ProQuest. The date of the search was July 19, 2025. This meta-analysis imposed no linguistic constraints. We utilized the Medical Subject Headings term “Neoplasms” [MeSH] for retrieval in the PubMed database. Due to the lack of a relevant subject term for “cadonilimab”, the search strategy for this intervention was established as “((cadonilimab[Title/Abstract]) OR (AK104[Title/Abstract])) OR (PD-1/CTLA-4[Title/Abstract]) “. For more information on the search strategies used for each database, please check out the **Supplementary Tables 1**–**7**.

### 2.2 Literature selection criteria

The inclusion criteria were formulated strictly following the PICOS framework: (1) The study population consisted of patients with histopathologically verified malignant solid tumors (Population, P); (2) Interventions involved cadonilimab monotherapy or cadonilimab in conjunction with other treatment protocols (Intervention, I). (3) Study designs encompassed randomized controlled trials (RCTs), prospective single-arm clinical trials, or prospective cohort studies (study design, S). The exclusion criteria included (1) patients with hematologic malignancies; (2) interventions involving immunotherapies other than cadonilimab, where specific data on cadonilimab could not be obtained; (3) study types such as retrospective studies, case reports, animal studies, or review articles; and (4) studies that did not provide extractable efficacy or safety outcome data. Comparison (C): The present study is chiefly comprised of single-arm trials; consequently, an internal control group was not included. Outcome (O): For further details, please refer to the information under Data extraction.

All literature screening was performed collaboratively by two researchers (MXD and TF). In cases of divergence, the final decision on inclusion was made after consultation with all participating researchers.

### 2.3 Data extraction

The extracted study details include first author, publication year, study design type, study name, NCT registration number, tumor type, number of enrolled patients, patient age, PD-1/PD-L1 expression status, cadonilimab dosage, and specific description of combination therapy regimens. For efficacy assessment, key metrics to extract include ORR, DCR, OS, and PFS. For safety information, the following should be extracted: incidence rates of AEs of all grades, incidence rates of Grade 1–2 AEs, incidence rates of Grade ≥3 AEs, and specific occurrences of common AEs such as anemia, neutropenia, thrombocytopenia, diarrhea, rash, mucocutaneous inflammation, hypertension, and thyroid dysfunction.

### 2.4 Quality assessment

As the included studies comprised both randomized controlled trials (RCTs) and single-arm studies, Version 2 of the Cochrane Risk of Bias Assessment Tool for Randomized Trials (RoB 2.0) was selected for the quality assessment of RCTs (25). The Methodological Index for Non-Randomized Studies (MINORS) was used to assess the quality of the single-arm clinical trials (26). Two researchers (TF and MXD) independently assessed the quality of each study. Where discrepancies arose, a third party (LT) was consulted to facilitate joint discussion and confirm the final results.

### 2.5 Statistical analysis

The statistical analysis of the study data was performed using R version 4.4.2. For single-group rate analysis, a generalized linear mixed model (GLMM) was employed and calculated using the ‘metaprop’ function in the ‘meta’ package. When pooling mPFS and mOS, a normality test was performed based on the known mPFS and mOS values. Where the data conformed to a normal distribution, the standard error was calculated using the formula SE = (upper_ci – lower_ci)/(2×1.96), and the pooled analysis was performed using the metamean function in the meta package. Heterogeneity was assessed using Cochran’s Q test and the I² statistic. As the I² value typically exceeds 90% in single-group meta-analyses, this study uniformly employed a random-effects model for the meta-analysis to ensure the robustness and reliability of the results (27). The results of the meta-analysis were illustrated using a forest plot, and the stability of the results was assessed using sensitivity analyses with stepwise exclusion. The Egger test was used to assess publication bias.

## 3 Results

### 3.1 Retrieved result

A comprehensive search of seven major databases yielded a total of 3101 relevant articles. Following the removal of duplicates, the total number of articles was reduced to 1435. Subsequent review of the abstracts resulted in the exclusion of 1074 articles, leaving 361 articles for full-text screening. Following a comprehensive evaluation of the full texts, 13 articles were found to satisfy the stipulated inclusion criteria (refer to **Supplementary Figure 1** for a detailed description of the screening process). All the studies included in this analysis were designed prospectively, comprising eleven single-arm clinical trials (15, 17, 19, 23, 28–34) and two randomized controlled trials (16, 18) (see **Supplementary Table 8** for specific baseline characteristics). Thirteen studies enrolled a total of 1359 patients with solid tumors, including 437 cases of gastric cancer or adenocarcinoma of the gastro-esophageal junction (G/GEJ), 382 cases of cervical cancer, 145 cases of non-small cell lung cancer (NSCLC), 83 cases of hepatocellular carcinoma, 48 cases of nasopharyngeal carcinoma, 22 cases of esophageal squamous cell carcinoma, and 242 cases of other unclassified solid tumors. Of these patients, 1331 were included in the efficacy analysis, and 1359 were included in the safety analysis. The number of patients included in the efficacy analysis was slightly lower than in the safety analysis. This discrepancy was because some patients in five of the studies received cadonilimab but were excluded from the efficacy analysis due to factors such as an absence of measurable lesions at baseline, a lack of post-treatment imaging assessments, failure to meet the eligibility criteria, a history of prior treatment, or failure to complete the prescribed treatment cycles (16, 23, 28, 30, 32).

### 3.2 Quality assessment

Two researchers assessed the risk of bias in 11 single-arm clinical trials using the MINORS tool and in two RCTs using the RoB 2.0 tool. Two researchers conducted these assessments independently. Where there was disagreement, a third researcher was consulted for a joint assessment. Ultimately, two of the 11 single-arm clinical trials were assessed as having a moderate risk of bias, two RCTs were assessed as having a low risk of bias, and nine were assessed as having a low risk of bias. The specific scores are shown in **Supplementary Table 9**.

### 3.3 Efficacy analysis

Of the 13 selected studies, all reported ORR, and 12 reported DCR. The pooled analysis yielded a combined ORR of 0.45 (95% confidence interval [CI]: 0.31–0.59) and a combined DCR of 0.84 (95% CI: 0.71–0.94). With regard to survival analysis, eight studies reported complete mPFS, and four reported complete mOS. The pooled analysis yielded a mPFS of 7.47 months (95% CI: 4.98–9.97) and a mOS of 12.89 months (95% CI: 10.09–15.68). Forest plots of pooled effect sizes for each efficacy endpoint are presented in **Supplementary Figure 2**.

### 3.4 Subgroup analysis

**Figure 1:**
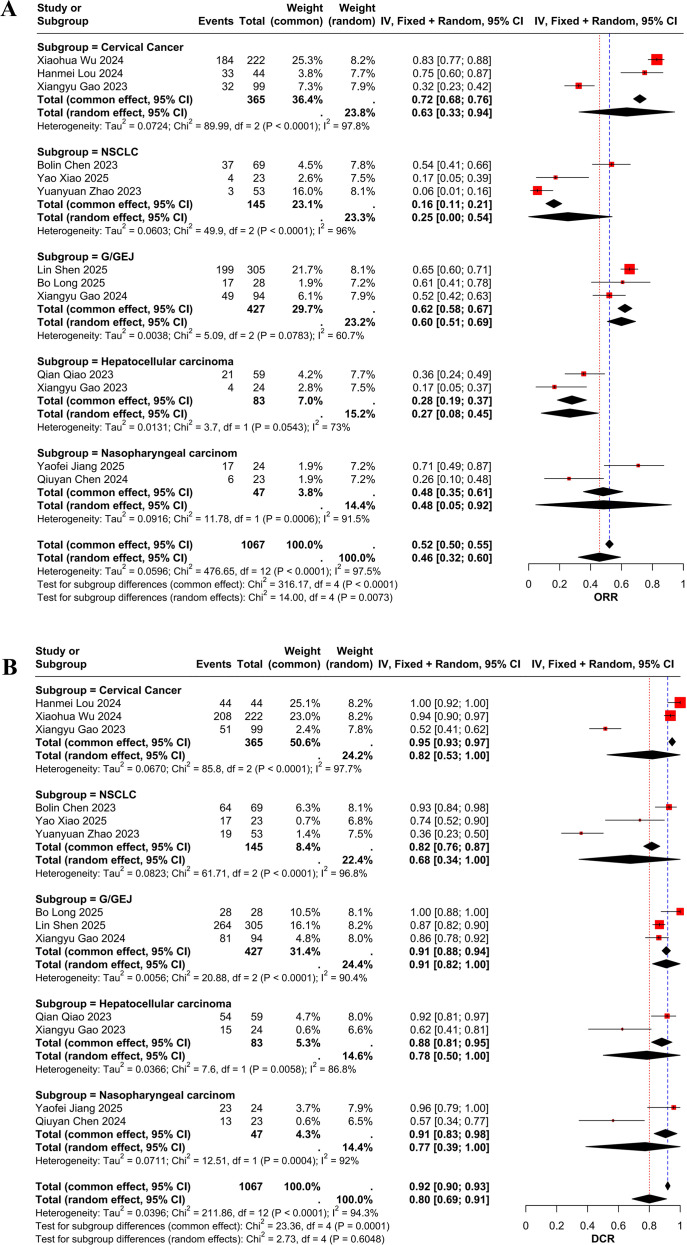
Forest plot showing subgroup analysis of ORR **(A)** and DCR **(B)** in different types of tumor groups. NSCLC denotes non-small-cell lung cancer, G/GEJ gastric or gastroesophageal junction adenocarcinoma.

A comprehensive analysis of 13 included studies was conducted, and on this basis, subgroup analyses were carried out. These analyses were based on different solid tumor types, the dosage of cadonilimab, and combination therapy regimens. The results demonstrated that, in terms of ORR, cervical cancer had the highest pooled effect size at 0.63 (95% CI: 0.33–0.94), followed by G/GEJ (0.60, 95% CI: 0.51–0.69), nasopharyngeal carcinoma (0.48, 95% CI: 0.05–0.92), hepatocellular carcinoma (0.27, 95% CI: 0.08–0.45) and NSCLC (0.25, 95% CI: 0.00–0.54) (**Figure 1A**). Following the application of a random-effects model, there was a statistically significant difference in ORR values across different tumor subgroups (p = 0.073 < 0.05). With regard to DCR, the pooled DCR was highest for G/GEJ cancer at 0.91 (95% CI: 0.82–1.00), followed by cervical cancer (0.82, 95% CI: 0.53–1.00), hepatocellular carcinoma (0.78, 95% CI: 0.50–1.00), nasopharyngeal carcinoma (0.77, 95% CI: 0.39–1.00) and NSCLC (0.68, 95% CI: 0.34–1.00) (**Figure 1B**). Following the application of a random-effects model, there were no statistically significant differences in DCR values across the different tumor type subgroups (p = 0.6048 > 0.05).

**Figure 2:**
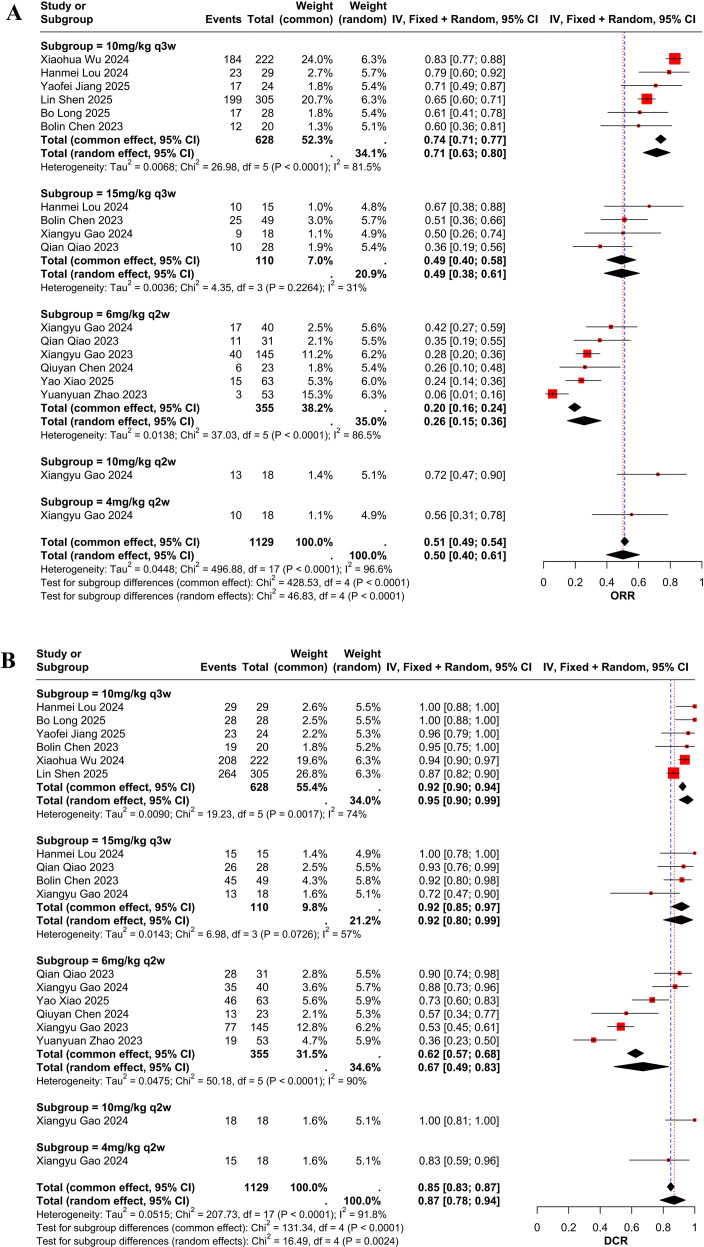
Forest plot showing subgroup analysis of ORR **(A)** and DCR **(B)** 0.85 [0.83; 0.87] 0.87 [0.78; 0.94] 0 0.2 0.4 0.6 0.8 1 DCR in different drug dosages.

Subgroup analyses based on cadonilimab dosage revealed differences in ORR and DCR across dose regimens. For ORR, the 10 mg/kg every 3 weeks (q3w) regimen demonstrated the highest pooled ORR of 0.71 (95% CI: 0.63–0.80). The 15 mg/kg q3w regimen was followed by a pooled ORR of 0.49 (95% CI: 0.38–0.61). The 6 mg/kg q2w regimen demonstrated the lowest pooled ORR at 0.26 (95% CI: 0.15–0.36), as detailed in **Figure 2A**. With regard to DCR, the 10 mg/kg q3w regimen once again exhibited the highest pooled DCR at 0.95 (95% CI: 0.90–0.99). The combined DCR for the 15 mg/kg q3w regimen was 0.92 (95% CI: 0.80–0.99). The combined DCR for the 6 mg/kg q2w regimen was comparatively lower at 0.67 (95% CI: 0.49–0.83), as illustrated in **Figure 2B**.

**Figure 3:**
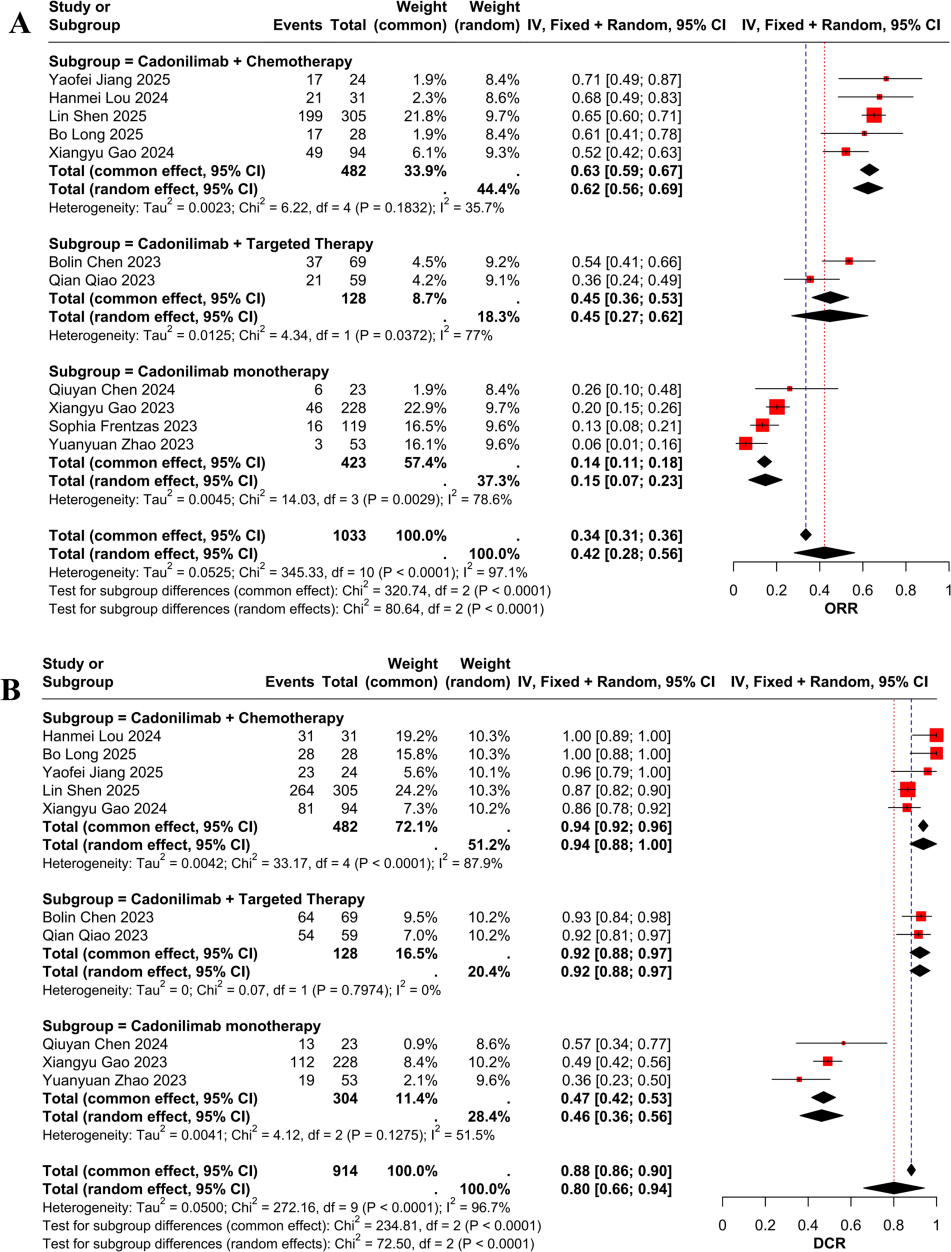
Forest plot showing subgroup analysis of ORR **(A)** and DCR **(B)** in different treatment options.

Subgroup analysis based on combination therapy regimens revealed significant differences in ORR and DCR across treatment strategies. Regarding ORR, cadonilimab combined with chemotherapy demonstrated the highest efficacy, achieving a pooled ORR of 0.62 (95% CI: 0.56–0.69). The combined ORR for cadonilimab plus targeted therapy was 0.45 (95% CI: 0.27–0.62). Cadonilimab monotherapy yielded the lowest combined ORR at 0.15 (95% CI: 0.07–0.23), as detailed in **Figure 3A**. Regarding DCR, the combination chemotherapy regimen again demonstrated the most significant efficacy, achieving a pooled DCR of 0.94 (95% CI: 0.88–1.00). The pooled DCR for the combination targeted therapy regimen was 0.92 (95% CI: 0.88–0.97). In contrast, the pooled DCR for monotherapy was 0.46 (95% CI: 0.36–0.56), significantly lower than that of combination regimens, as shown in **Figure 3B**.

### 3.5 Safety analysis

**Figure 4:**
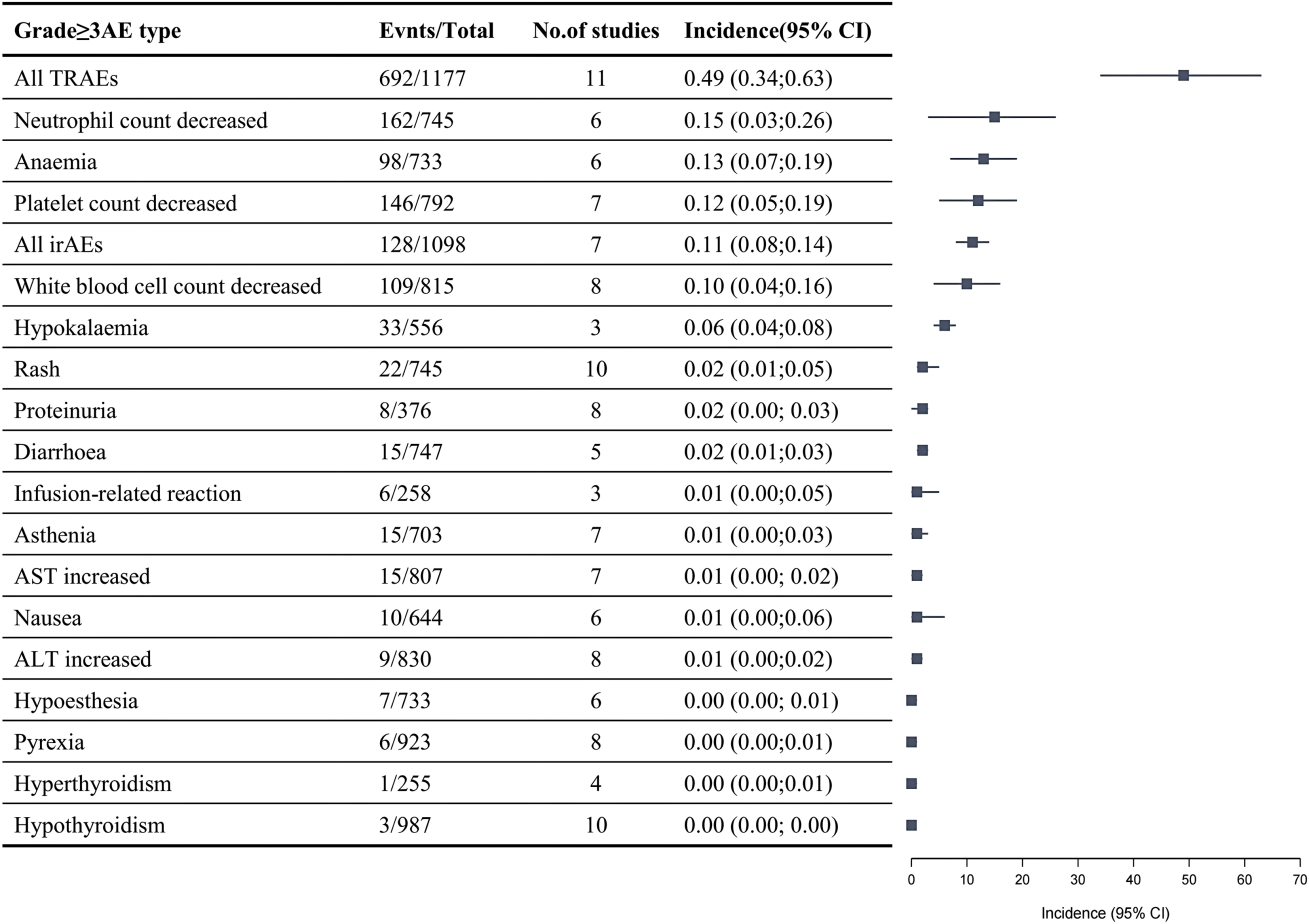
Forest plots show the pooled incidence of various grade > 3 adverse events. ALT denotes alanine aminotransferase, and AST aspartate aminotransferase.

A pooled analysis of AEs across the 13 included studies demonstrated an incidence rate of 0.99 (95% CI: 0.98–1.00) for AEs of any grade. The incidence rate of TRAEs of grade 3 or higher was 0.49 (95% CI: 0.34–0.63), while the incidence rate of irAEs of grade 3 or higher was 0.11 (95% CI: 0.08–0.14). Among Grade 3 or higher TRAEs, the most prevalent was neutropenia, with an incidence rate of 0.15 (95% CI: 0.03–0.26). The remaining incidence rates, in descending order, were anemia 0.13 (95% CI: 0.07–0.19), thrombocytopenia 0.12 (95% CI: 0.05–0.19), leukopenia 0.10 (95% CI: 0.04–0.16), hypokalemia 0.06 (95% CI: 0.04–0.08), and rash 0.02 (95% CI: 0.01–0.05), as illustrated in **Figure 4**.

### 3.6 Sensitivity analysis and assessment of publication bias

Heterogeneity tests were conducted for the efficacy endpoints included in the study (ORR, DCR, mPFS, and mOS). The I^2^ statistic exceeded 50% for all indicators, indicating substantial heterogeneity among the studies included in this meta-analysis. This heterogeneity is presumably primarily attributable to the fact that the majority of the included studies were single-arm clinical trials. Subsequent sensitivity analysis demonstrated that the pooled effect size remained stable after the sequential exclusion of each individual study, thereby indicating robust consistency of the findings. Furthermore, the Egger test for publication bias yielded P-values exceeding 0.05 for all efficacy endpoints, suggesting that no significant publication bias was detected in this study.

## 4 Discussion

The notion of bsAbs was initially put forward by Alfred Nisonoff in the 1960s. He successfully engineered F(ab’)_2_ molecules with dual specificity and validated their dual-target binding capability (35). This field has since evolved into a major direction in tumor immunotherapy (36). In comparison with the limited efficacy of antibodies targeting a single immune checkpoint and its ligands, bsAbs offer several advantages. These include the ability to simultaneously inhibit the primary target and compensatory pathway targets, to block two pathways concurrently, and to prevent immune escape (37). A substantial number of clinical studies on immune checkpoint bsAbs are currently underway, with core targets including PD-1/PD-L1, PD-L1/CTLA-4, and PD-1/CD47 (38). Following years of development, multiple bsAbs have been developed as therapeutic agents for solid tumors and hematologic malignancies (39). Cadonilimab is the world’s first bispecific antibody drug that simultaneously targets PD-1 and CTLA-4. It has outstanding targeting of the tumor microenvironment, can significantly reduce off-target damage to normal tissues, and effectively avoids the problem of adverse reactions caused by combining monoclonal antibodies, thus balancing the high efficiency and safety of treatment (10). Numerous completed or ongoing clinical trials have been conducted on the use of cadonilimab for treating solid tumors, with preliminary results suggesting its promising anticancer efficacy. Nonetheless, current research exhibits deficiencies regarding the amalgamation of efficacy data, the thoroughness of subgroup analysis, and the methodical approach to safety evaluations. A comprehensive, cross-tumor-type systematic review is also absent. This meta-analysis, for the first time, consolidates data from 13 clinical studies involving 1359 patients, thoroughly assessing the efficacy and safety profiles of cadonilimab across various solid tumor types and contrasting the clinical benefits of different treatment regimens, thereby addressing existing gaps in clinical evidence.

The pooled analysis results of this study indicate that cadonilimab has beneficial anti-tumor efficacy and survival advantages in solid malignancies. The ORR was 45%, while the DCR was 84%. In the context of survival analysis, the combined mPFS was 7.47 months, while the mOS was 12.89 months. The results above indicate that cadonilimab demonstrates a fair disease control rate and moderate objective response rate across multiple solid tumors while also achieving prolonged survival. As a uniquely structured tetravalent PD-1/CTLA-4 bispecific antibody, cadonilimab has been demonstrated to comprehensively reverse T-cell exhaustion in the tumor microenvironment, alleviate immune suppression, and reduce non-specific immune activation in peripheral tissues (40–42). In comparison with combination regimens of PD-1/CTLA-4 monoclonal antibodies, this drug has been shown to significantly reduce off-target effects while maintaining anti-tumor efficacy. This approach presents a novel, effective alternative for immunotherapy across a range of tumor types, particularly for patients exhibiting a poor response to single-target ICI therapy (40, 43, 44). However, this meta-analysis exhibits significant clinical heterogeneity in its results, arising from factors such as different tumor types, the number of treatment lines, cadonilimab dosing regimens and monotherapy versus various combination regimens. The overall combined ORR and DCR are broad summary measures only; subgroup analysis results may provide a more accurate picture. As the majority of the 13 studies included in this meta-analysis reported only mPFS and mOS and 95% CIs and lacked key data such as hazard ratios (HRs), it was not possible to conduct a standard HR-based survival meta-analysis. This study therefore only pooled median survival times and 95% CIs; this method cannot replace classical HR analysis, makes it difficult to precisely quantify the relative risk of prognosis, and can only provide a rough indication of survival levels. The pooled survival results in this study are exploratory in nature and are subject to methodological limitations; therefore, caution is advised in their clinical interpretation and application.

This study further evaluated the therapeutic efficacy of cadonilimab across different tumor types through subgroup analysis. The results demonstrated that its ORR and DCR were significantly superior in cervical cancer, G/GEJ compared to hepatocellular carcinoma, nasopharyngeal carcinoma, and NSCLC. The observed variations in efficacy may be attributed to differences in tumor mutational burden (TMB), tumor-infiltrating lymphocyte (TIL) levels, PD-1/CTLA-4 expression levels, and tumor mismatch repair (MMR) status across different tumors (15, 34, 45). Furthermore, the number of treatment lines and heterogeneity in combination regimens across tumor types may also influence treatment outcomes (45). Based on the findings of this study and the existing literature, it can be inferred that cadonilimab has considerable potential for the clinical treatment of cervical cancer and G/GEJ (46–48).

In the first-in-human clinical study of cadonilimab, unambiguous objective antitumor responses were identified exclusively at dosage levels that exceeded 2.0 mg/kg. No linear positive correlation between dose and overall efficacy metrics was identified, though differences in efficacy performance were noted across different dose cohorts. The 6 mg/kg q2w regimen exhibited an optimal balance between efficacy and safety, aligning with the approved recommended dose (10, 15). Subsequent extensive clinical studies further validated the dose-response relationship. This study’s findings indicated that the 10 mg/kg q3w regimen exhibited a higher ORR and DCR in comparison to both the 15 mg/kg q3w and 6 mg/kg q2w regimens. Based on the data from this meta-analysis, the 10 mg/kg Q3W regimen showed a trend towards relatively superior efficacy.

Studies indicate that the antitumor activity of cadonilimab combination therapy outperforms monotherapy. This advantage has been consistently demonstrated across different tumor types and treatment lines, aligning with the findings of this study (10). The prevailing combination therapy strategies currently in use principally consist of combination chemotherapy, combination chemotherapy with anti-angiogenic agents, and combination with targeted therapies (45). The enhanced efficacy of combination regimens can be attributed to their synergistic effects in reshaping the tumor microenvironment and activating anti-tumor immune responses. Furthermore, monotherapy regimens have exhibited antitumor activity in patients with advanced recurrent or previously PD-1/PD-L1 therapy-failed solid tumors (28, 29, 44, 45, 49).

The safety analysis results of this study demonstrate that the incidence rate of AEs of all grades was 99%, while the incidence rate of TRAEs of grade ≥3 was 49%. Among these, grade ≥3 TRAEs primarily manifested as chemotherapy-related hematologic toxicity rather than immune-related toxicity caused by the bsAbs itself, suggesting that toxic reactions mainly originated from the combination chemotherapy drugs used in the combination therapy. In this study, the incidence of ≥ Grade 3 irAEs was only 11%, which is significantly lower than that observed with traditional PD-1/CTLA-4 combination therapy regimens (50–52). Research indicates that the superior safety profile of bsAbs compared to traditional PD-1/CTLA-4 combination regimens may stem from their unique molecular structure. By targeting multiple immune checkpoints, bsAbs mitigate immunosuppressive phenotypes and effectively circumvent immune tolerance within the tumor microenvironment (6, 53). In addition, bsAbs offer a distinct advantage, as they are capable of cross-linking two distinct cell types or binding two molecules present cis on the same cell membrane. By modulating the binding affinities of the two binding sites, bsAbs can mitigate off-target effects in normal tissues and reduce TRAEs (40, 54).

This meta-analysis has the following limitations: Firstly, the majority of the 13 included studies were single-arm clinical trials, with only two randomized controlled trials. Consequently, there is a lack of data from strictly controlled groups, and no quantitative comparison of the efficacy and safety of cadonilimab versus other treatment regimens has been conducted. Including data from a large number of single-arm trials increases the heterogeneity of the study and the risk of publication bias (55). Secondly, the analysis was based solely on pooled data from individual trials, without access to individual-level data. This limited the possibility of exploring the impact of patients’ baseline characteristics on efficacy. Thirdly, sample sizes for certain tumor types (e.g., nasopharyngeal carcinoma and esophageal squamous cell carcinoma) were small, reducing the accuracy of the results. Fourthly, follow-up periods in some studies were short, resulting in a lack of long-term prognostic indicators such as mPFS and mOS. This limits in-depth analysis of long-term efficacy and survival characteristics. Fifth, the pooled results of this study may be subject to significant bias due to factors such as different tumor types, treatment lines, drug dosages, treatment regimens, and disease contexts.

## 5 Conclusion

This meta-analysis indicates that cadonilimab exhibits good antitumor activity in patients with various solid tumors and is well tolerated. Its efficacy may be superior in cervical cancer, gastric cancer and G/GEJ compared with hepatocellular carcinoma, nasopharyngeal carcinoma and NSCLC. The 10 mg/kg q3w dosing regimen may be superior to the 15 mg/kg q3w and 6 mg/kg q2w regimens. Treatment regimens combining cadonilimab with chemotherapy demonstrated superior efficacy compared to monotherapy. This study provides evidence-based support for the clinical application of cadonilimab; however, as it included a large number of single-arm trials and the sample sizes for some tumor types were limited, large-scale RCTs are still required in the future to validate its long-term efficacy and safety and to identify biomarkers associated with treatment response.

## Data Availability

The original contributions presented in the study are included in the article/**Supplementary Material**. Further inquiries can be directed to the corresponding author.
