# Supplementary material for: Efficacy and safety of cadonilimab for malignant solid tumor treatment: a systematic review and meta-analysis
Source: Front Immunol. 2026 May 29;17:1851837. doi: 10.3389/fimmu.2026.1851837 (PMC13260096; doi:10.3389/fimmu.2026.1851837)
Supplement: Supplementary file 1 [file DataSheet1.docx]

Supplementary Material

# Supplementary Figures and Tables

## Supplementary Figures

##
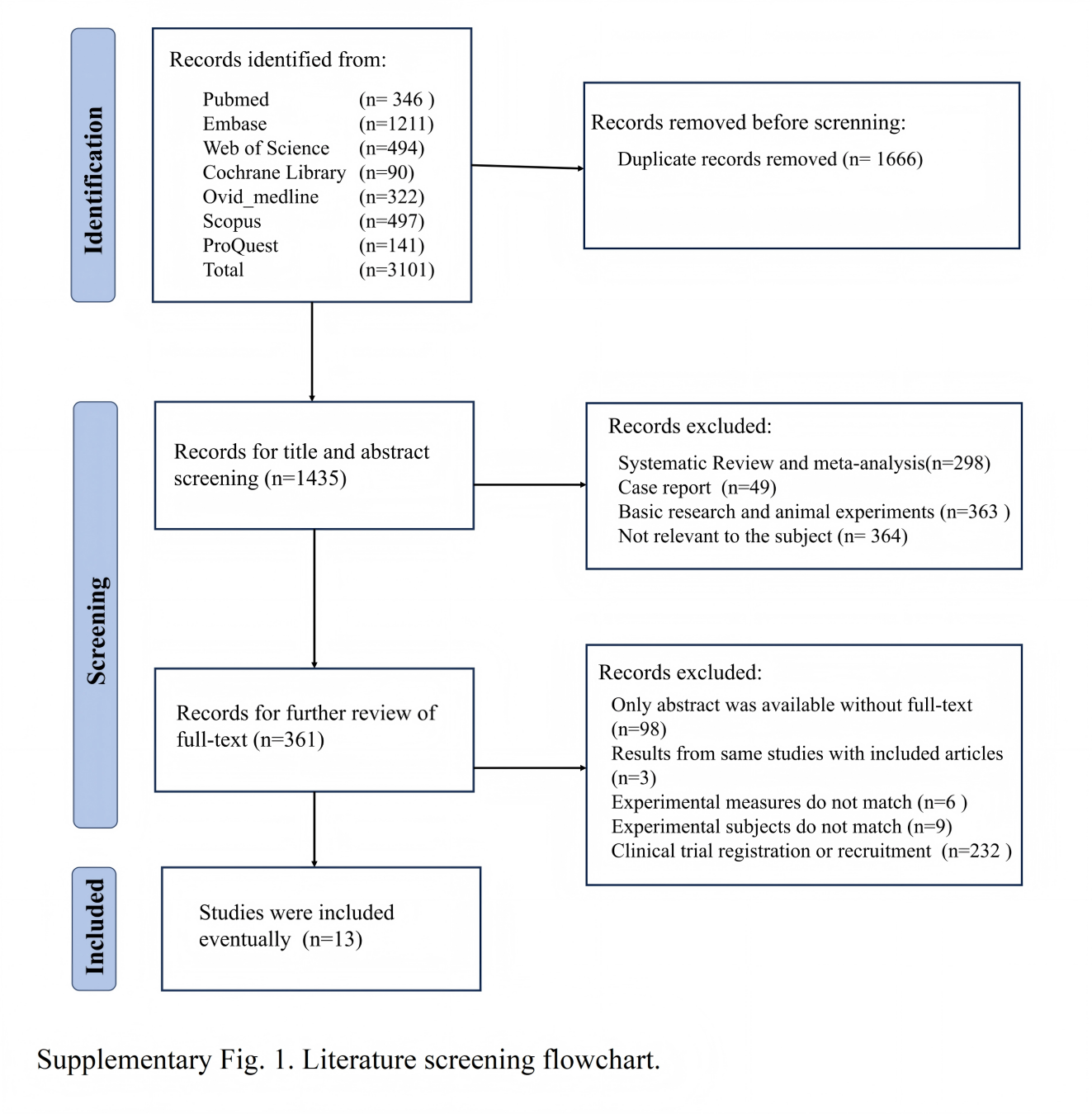


## Supplementary Figures 1 Literature screening flowchart.


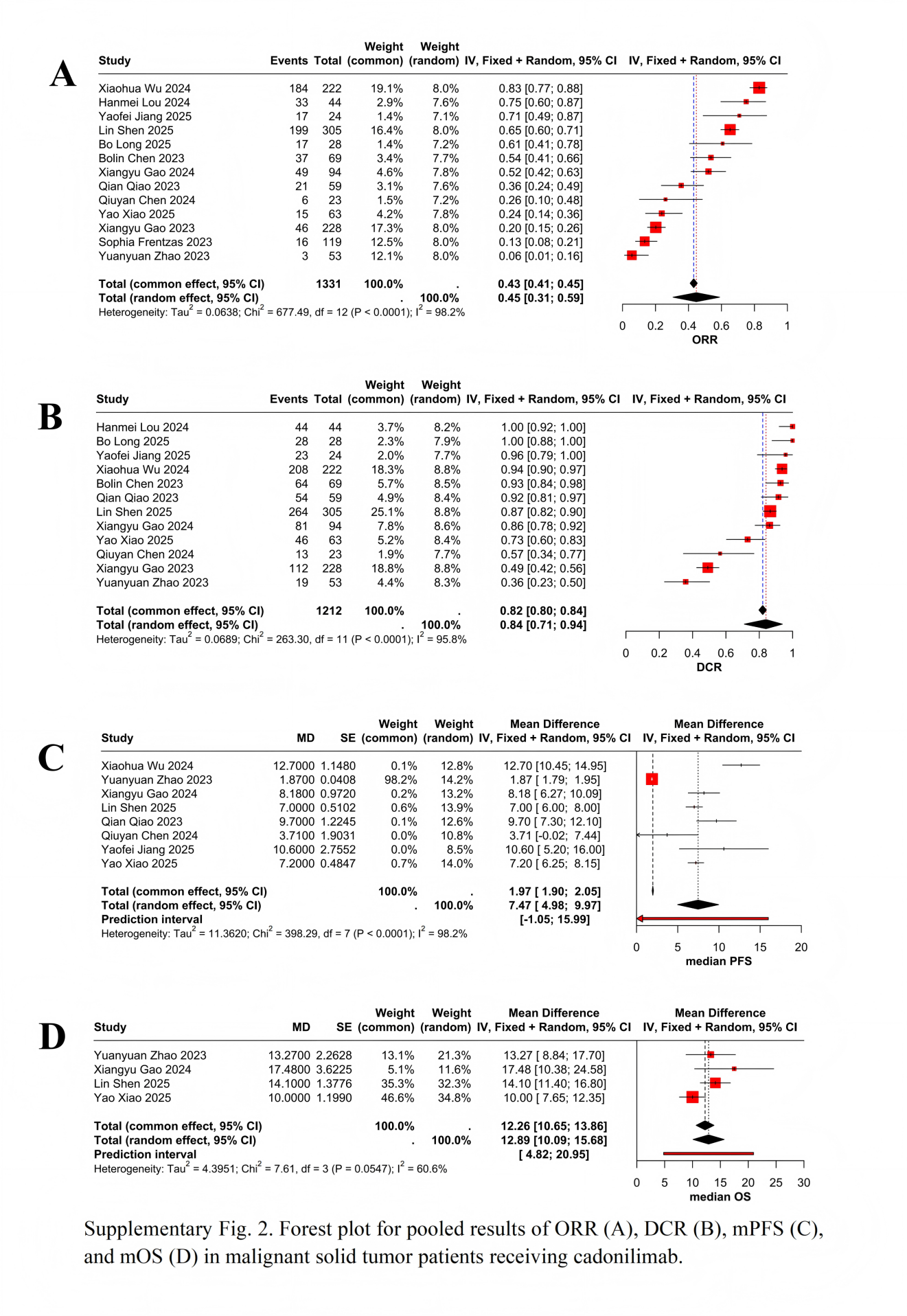


**Supplementary Figures 2.** Forest plot for pooled results of ORR (A), DCR (B), mPFS (C).and mOS (D) in malignant solid tumor patients receiving cadonilimab.

## Supplementary Tables

**Supplementary Table 1** Search strategy in PubMed.

| **Search** | **Query** | **Number of search results 2025.07.19** | **Remarks** |
| --- | --- | --- | --- |
| #1 | "Neoplasms"[Mesh] | 4,132,105 |  |
| #2 | ((((((((((((((((Tumors[Title/Abstract]) OR (Neoplasia[Title/Abstract])) OR (Neoplasias[Title/Abstract])) OR (Neoplasm[Title/Abstract])) OR (Tumor[Title/Abstract])) OR (Cancer[Title/Abstract])) OR (Cancers[Title/Abstract])) OR (Malignant Neoplasm[Title/Abstract])) OR (Malignancy[Title/Abstract])) OR (Malignancies[Title/Abstract])) OR (Malignant Neoplasms[Title/Abstract])) OR (Neoplasm, Malignant[Title/Abstract])) OR (Neoplasms, Malignant[Title/Abstract])) OR (Benign Neoplasms[Title/Abstract])) OR (Neoplasms, Benign[Title/Abstract])) OR (Neoplasm, Benign[Title/Abstract])) OR (Benign Neoplasm[Title/Abstract]) | 3,828,659 |  |
| **#3** | **#1 OR #2** | 5,362,136 | P |
| #4 | ((cadonilimab[Title/Abstract]) OR (AK104[Title/Abstract])) OR (PD-1/CTLA-4[Title/Abstract]) | 432 | I |
| **#5** | **#3 AND #4** | 346 | P and I |

P: Participant; I: Intervention.

**Supplementary Table 2** Search strategy in Embase.

| **Search** | **Query** | **Number of search results 2025.07.19** | **Remarks** |
| --- | --- | --- | --- |
| #1 | 'neoplasm'/exp | 6,812,447 |  |
| #2 | 'tumors':ab,ti OR 'neoplasia':ab,ti OR 'neoplasias':ab,ti OR 'neoplasm':ab,ti OR 'tumor':ab,ti OR 'cancer':ab,ti OR 'cancers':ab,ti OR 'malignant neoplasm':ab,ti OR 'malignancy':ab,ti OR 'malignancies':ab,ti OR 'malignant neoplasms':ab,ti OR 'neoplasm, malignant':ab,ti OR 'neoplasms, malignant':ab,ti OR 'benign neoplasms':ab,ti OR 'neoplasms, benign':ab,ti OR 'neoplasm, benign':ab,ti OR 'benign neoplasm':ab,ti | 5,374,504 |  |
| #3 | **#1 OR #2** | 7,802,081 | P |
| #4 | 'cadonilimab'/exp | 467 |  |
| #5 | 'ak104':ab,ti OR 'pd-1/ctla-4':ab,ti | 1,089 |  |
| #6 | **#4 OR #5** | 1,346 | I |
| #7 | **#3 AND #6** | 1,211 | P and I |

**Supplementary Table 3** Search strategy in Web of Science.

| **Search** | **Query** | **Number of search results 2025.07.19** | **Remarks** |
| --- | --- | --- | --- |
| #1 | Neoplasms (Topic) or Tumors (Topic) or Neoplasia (Topic) or Neoplasias (Topic) or Neoplasm (Topic) or Tumor (Topic) or Cancer (Topic) or Cancers (Topic) or Malignant Neoplasm (Topic) or Malignancy (Topic) or Malignancies (Topic) or Malignant Neoplasms (Topic) or Neoplasm, Malignant (Topic) or Neoplasms, Malignant (Topic) or Benign Neoplasms (Topic) or Neoplasms, Benign (Topic) or Neoplasm, Benign (Topic) or Benign Neoplasm (Topic) and Preprint Citation Index (Exclude – Database) | 7,850,802 | P |
| #2 | cadonilimab (Topic) or AK104 (Topic) or PD-1/CTLA-4 (Topic) and Preprint Citation Index (Exclude – Database) | 619 | I |
| **#3** | **#1 AND #2** | 494 | P and I |

**Supplementary Table 4** Search strategy in Cochrane Library.

| **Search** | **Query** | **Number of search results 2025.07.19** | **Remarks** |
| --- | --- | --- | --- |
| #1 | MeSH descriptor: [Neoplasms] explode all trees | 128,502 |  |
| #2 | 'tumors':ab,ti OR 'neoplasia':ab,ti OR 'neoplasias':ab,ti OR 'neoplasm':ab,ti OR 'tumor':ab,ti OR 'cancer':ab,ti OR 'cancers':ab,ti OR 'malignant neoplasm':ab,ti OR 'malignancy':ab,ti OR 'malignancies':ab,ti OR 'malignant neoplasms':ab,ti OR 'neoplasm, malignant':ab,ti OR 'neoplasms, malignant':ab,ti OR 'benign neoplasms':ab,ti OR 'neoplasms, benign':ab,ti OR 'neoplasm, benign':ab,ti OR 'benign neoplasm':ab,ti | 269,865 |  |
| **#3** | **#1 OR #2** | 294,336 | P |
| #4 | (cadonilimab):ab,ti,kw OR (AK104):ab,ti,kw OR (PD-1/CTLA-4):ab,ti,kw | 99 | I |
| **#5** | **#3 AND #4** | 90 | P and I |

**Supplementary Table 5** Search strategy in Ovid Medline.

| **Search** | **Query** | **Number of search results 2025.07.19** | **Remarks** |
| --- | --- | --- | --- |
| #1 | (Neoplasms or Tumors or Neoplasia or Neoplasias or Neoplasm or Tumor or Cancer or Cancers or Malignant Neoplasm or Malignancy or Malignancies or Malignant Neoplasms or Neoplasm, Malignant or Neoplasms, Malignant or Benign Neoplasms or Neoplasms, Benign or Neoplasm, Benign).ti,ab,kw. | 3,806,902 | P |
| #2 | (cadonilimab OR AK104 OR PD-1/CTLA-4).ti,ab,kw. | 8,902 | I |
| **#3** | **#1 OR #2** | 322 | P and I |

**Supplementary Table 6** Search strategy in ProQuest.

| **Search** | **Query** | **Number of search results 2025.07.19** | **Remarks** |
| --- | --- | --- | --- |
| #1 | ABSTRACT，TITLE（Neoplasms OR Tumors OR Neoplasia OR Neoplasias OR Neoplasm OR Tumor OR Cancer OR Cancers OR Malignant Neoplasm OR Malignancy OR Malignancies OR Malignant Neoplasms OR Neoplasm, Malignant OR Neoplasms, Malignant OR Benign Neoplasms OR Neoplasms, Benign OR Neoplasm, Benign OR Benign Neoplasm） | 5,062,837 | P |
| #2 | ABSTRACT，TITLE（ "PD-1/CTLA-4" ） | 15 |  |
| **#3** | **ABSTRACT**，TITLE（cadonilimab or AK104） | 242 |  |
| **#4** | **#2 OR #3** | 257 | I |
| **#5** | **#1 AND #4** | 195 | P and I |
| **#6** | **Exclude:Publication Type: Company News; Books** | 141 | P and I |

**Supplementary Table 7** Search strategy in Scopus.

| **Search** | **Query** | **Number of search results 2025.07.19** | **Remarks** |
| --- | --- | --- | --- |
| #1 | ( TITLE-ABS-KEY ( "Neoplasms" OR "Tumors" OR "Neoplasia" OR "Neoplasias" OR "Neoplasm" OR "Tumor" OR "Cancer" OR "Cancers" OR "Malignant Neoplasm" OR "Malignancy" OR "Malignancies" OR "Malignant Neoplasms" OR "Neoplasm, Malignant" OR "Neoplasms, Malignant" OR "Benign Neoplasms" OR "Neoplasms, Benign" OR "Neoplasm, Benign" OR "Benign Neoplasm" ) AND TITLE-ABS-KEY ( "cadonilimab" OR "AK104" OR "PD-1/CTLA-4" ) ) | 497 | P and I |

**Supplementary Table 8** Basic information on the studies.

| **First author** | **Publication year** | **Trial Abbreviated Name** | **Registration number** | **Nation** | **Trial phase** | **Intervention** | **Cadonilimab usage and dosage** | **Sample**  **size** | **Cancer types** |
| --- | --- | --- | --- | --- | --- | --- | --- | --- | --- |
| Xiaohua Wu | 2024 | COMPASSION-16 | NCT04982237 | China | RCT | Cadonilimab+chemotherapy±Bev | 10mg/kg q3w | 226 | cervical cancer |
| Xiangyu Gao | 2023 | COMPASSION-03 | NCT03852251 | China | Single-arm | Cadonilimab monotherapy | 6mg/kg q2w；  10mg/kg q2w；  450mg q2w； | 240 | solid tumours |
| Yuanyuan Zhao | 2023 | AK104-202 study | NCT04172454 | China | Single-arm | Cadonilimab monotherapy | 6mg/kg q2w | 53 | NSCLC |
| Hanmei Lou | 2024 | COMPASSION-13 | NCT04868708 | China | Prospective cohort study | Cadonilimab+chemotherapy±Bev | 10mg/kg q3w；  15mg/kg q3w； | 45 | cervical cancer |
| Xiangyu Gao | 2024 | COMPASSION-04 | CTR20182027 | China | Single-arm | Cadonilimab+chemotherapy | 4mg/kg q2w；  6mg/kg q2w；  10mg/kg q2w；  15mg/kg q3w； | 94 | G/GEJ |
| Lin Shen | 2025 | COMPASSION-15 | NCT05008783 | China | RCT | Cadonilimab+chemotherapy | 10mg/kg q3w； | 305 | G/GEJ |
| Sophia Frentzas | 2023 | COMPASSION-01 | NCT03261011 | Australia and China | Single-arm | Cadonilimab monotherapy | / | 119 | solid tumours |
| Bo Long | 2025 | / | ChiCTR2200066893 | China | Single-arm | Cadonilimab+chemotherapy | 10mg/kg q3w； | 38 | G/GEJ |
| Qian Qiao | 2023 | COMPASSION-08 | NCT04444167 | China | Single-arm | Cadonilimab+targeted therapy | 6mg/kg q2w；  15mg/kg q3w； | 59 | Hepatocellular carcinoma |
| Qiuyan Chen | 2024 | COMPASSION-06 | NCT04220307 | China | Single-arm | Cadonilimab monotherapy | 6mg/kg q2w； | 23 | Nasopharyngeal carcinoma |
| Yaofei Jiang | 2025 | AK104-IIT-018 Study | ChiCTR2200067057 | China | Single-arm | Cadonilimab+chemotherapy | 6mg/kg q2w； | 25 | Nasopharyngeal carcinoma |
| Bolin Chen | 2023 | / | NCT04646330 | China | Prospective cohort study | Cadonilimab+targeted therapy | 10mg/kg q3w；  15mg/kg q3w； | 69 | NSCLC |
| Yao Xiao | 2025 | / | NCT05915481 | China | Single-arm | Cadonilimab+ radiotherapy | 6mg/kg q2w； | 63 | solid tumours |

NSCLC: non-small-cell lung cancer; G/GEJ: gastric or gastroesophageal junction adenocarcinoma.

**Supplementary Table 9**  Risk-of-bias assessment of the included studies according to the MINORS scale

| **Methodological items** | **Xiangyu Gao 2023** | **Yuanyuan Zhao 2023** | **Hanmei Lou 2024** | **Xiangyu Gao 2024** | **Sophia Frentzas 2023** | **Bo Long 2025** | **Qian Qiao 2023** | **Qiuyan Chen 2024** | **Yaofei Jiang 2025** | **Bolin Chen 2023** | **Yao Xiao 2025** |
| --- | --- | --- | --- | --- | --- | --- | --- | --- | --- | --- | --- |
| A clearly stated aim | 2 | 2 | 2 | 2 | 2 | 2 | 2 | 2 | 2 | 2 | 2 |
| Inclusion of consecutive patients | 2 | 2 | 2 | 1 | 2 | 2 | 2 | 2 | 1 | 1 | 1 |
| Prospective collection of data | 2 | 2 | 2 | 2 | 2 | 2 | 2 | 2 | 2 | 2 | 1 |
| Endpoints appropriate to the aim of the study | 2 | 2 | 2 | 2 | 2 | 2 | 2 | 2 | 2 | 2 | 2 |
| Unbiased assessment of the study endpoint | 1 | 1 | 1 | 1 | 1 | 2 | 1 | 1 | 1 | 1 | 1 |
| Follow-up period appropriate to the aim of the study | 2 | 2 | 2 | 2 | 2 | 1 | 2 | 2 | 2 | 2 | 2 |
| Loss to follow-up less than 5% | 2 | 2 | 2 | 1 | 2 | 2 | 2 | 2 | 2 | 2 | 2 |
| Prospective calculation of the study size | 2 | 2 | 2 | 1 | 2 | 2 | 2 | 1 | 2 | 2 | 0 |
| **Total score** | 15 | 15 | 15 | 12 | 15 | **15** | **15** | **15** | **15** | **13** | **12** |

The items are scored 0 (not reported), 1 (reported but inadequate) or 2 (reported and adequate)
